# 达芬奇机器人治疗肺孤立结节的临床体会

**DOI:** 10.3779/j.issn.1009-3419.2014.07.07

**Published:** 2014-07-20

**Authors:** 向东 童, 世广 许, 述民 王, 浩 孟, 昕 高, 洪 滕, 仁泉 丁, 星池 刘, 博 李, 惟 徐, 通 王

**Affiliations:** 110016 沈阳，沈阳军区总医院胸外科 Department of Thoracic Surgery, General Hospital of Shenyang Military Region, Shenyang 110016, China

**Keywords:** 孤立肺结节, 肺癌, 机器人, 达芬奇手术系统, Solitary pulmonary nodule, Lung neoplasms, Robotics, Da Vinci Surgical System

## Abstract

**背景与目的:**

肺孤立结节（solitary pulmonary nodule, SPN）定义为一个圆形的直径小于3 cm的肺实质内的病变，不伴有肺不张和淋巴结病变。本研究旨在探讨达芬奇机器人治疗SPN的临床体会。

**方法:**

2011年11月-2014年3月，沈阳军区总医院应用达芬奇机器人治疗SPN 9例，其中男性3例；女性6例；年龄41岁-74岁，平均（51±9.9）岁；患者多数无明显临床症状（健康体检发现7例，咳嗽咳痰2例）；病史时间4天-3年（中位数12个月）；病变均为周围型肺结节病灶，直径为0.8 cm-2.8 cm，平均（1.4±0.6）cm；术中切取病变送冰冻病理检查，证实为恶性病变者行肺叶切除或楔形切除并常规清除肺门和纵隔淋巴结。手术采用全麻、双腔管气管插管，患者健侧卧位，胸部垫高，双手屈曲抱枕于头前，折刀位。孔位为腋后线第8肋间为进镜孔，肩胛线第8肋间、腋前线与锁中线间第5肋间为器械孔，腋中线第7肋间为辅助口。

**结果:**

术后病理为良性病变4例（炎性假瘤3例，错构瘤1例），恶性病变5例，均为腺癌。手术包括楔形切除4例，右肺中叶切除+淋巴结清除术2例，左肺上叶切除+淋巴结清除术1例，其余2例肺癌患者因为心肺功能差，病变小于2 cm，行楔形切除+淋巴结清除术。9例均顺利完成机器人手术，所有患者无严重术后并发症，均顺利出院。随访时间为0.1个月-18.5个月（中位数11个月），无复发、转移。

**结论:**

SPN病变应该予以积极手术治疗，提高早期肺癌的诊断率和治愈率，达芬奇机器人手术对于SPN的治疗是一种安全、微创的手术方法，在SPN病变的诊治中具有较高的价值。

## Abstract (en)

**Background and objective:**

A solitary pulmonary nodule (SPN) is defined as a round intraparenchimal lung lesion less than 3 cm in size, not associated with atelectasis or adenopathy. The aim of this study is to learn clinical experience of the treatment of SPN with Da Vinci Surgical System.

**Methods:**

A total of 9 patients with solitary pulmonary nodules (SPN) less than 3 cm in diameter was treated with Da Vinci Surgical System (Intuitive Surgical, California) in thoracic surgery department from General Hospital of Shenyang Militrary Region from November 2011 to March 2014. This group of patients included 3 males and 6 females, and the mean age was 51±9.9 yr (range: 41-74 yr). Most of the patients were no obvious clinical symptoms (7 cases were found by physical examination, others were with cough and expectoration). Their median medical history was 12 mo (range: 4 d-3 yr). All the lesions of patients were peripheral pulmonary nodules and the mean diameter of those was (1.4±0.6) cm(range: 0.8-2.8 cm). Wedge-shaped resection or lobectomy was performed depending on the result of rapid pathology and systemic lymph node dissection was done for malignant leision. We used general anesthesis with double lumens trachea cannula. We set the patients in lateral decubitus position with jackknife. The patient cart enter from top of the patient. The position of trocars would be set according to the position of lesion. A 12 mm incision was positioned at the 8th intercostal space in the posterior axillary line as vision port, and two 8 mm incisions were positioned at the 5^th^ intercostal space between the anterior axillary line and midclavicular line, and the 8^th^ infrascapular line as robotic instrument ports about 10 cm apart from the vision port. One additional auxiliary small incision for instrument without retracting ribs was set at the 7^th^ intercostal space in the middle axillary line.

**Results:**

There were 4 benign leisions and 5 malignancies identified. Wedge-shaped resection was performed for 4 patients, lobectomy with systemic lymph node dissection for 3 patients (including 2 right middle lobectomies and 1 left upper lobectomy) and wedge-shaped resection with systemic lymph node dissection for 2 patients of poor lung function. All of the 9 cases were completed with total robotic procedure without conversion. The pathological results included 3 inflammatory pseudotumors, 1 hamartoma, 5 adenocarcinomas. All of the 29 patients were hospital discharged smoothly. The patients were followed up for 0.1-18.5 mo (median 11 mo) without recurrence or metastasis.

**Conclusion:**

The SPN patients should be given active surgical treatments to improve the diagnose rate as well as the cure rate of early non-small cell lung cancer. Since da Vinci Surgical System is a safe and minimally invasive treatment for SPN, it has higher value to the diagnosis and treatment of SPN.

肺孤立结节（solitary pulmonary nodule, SPN）定义为一个圆形的直径小于3 cm的肺实质内的病变，不伴有肺不张和淋巴结病变^[1]^。随着影像学技术的不断发展和健康体检的普及，越来越多的无症状的SPN病例被发现，直径大于2 cm者恶性肿瘤的发生率可高达64%-82%^[2]^，因此对这类病例的诊治，能大大提高肺癌的治愈率。近年来达芬奇机器人作为一种新兴的技术方法在胸外科领域得到越来越广泛的应用。沈阳军区总医院于2011年11月-2014年3月已完成达芬奇机器人手术294例，病种及手术包括纵隔肿瘤、食管平滑肌瘤切除术、肺楔形切除、肺叶切除术、肺癌根治术，其中9例符合SPN标准，现总结如下。

## 1 资料与方法

### 1.1 临床资料

本研究采用回顾性方法总结沈阳军区总医院2011年11月-2014年3月达芬奇机器人手术的9例SPN患者。其中男性3例，女性6例；年龄范围41岁-74岁，平均（51±9.9）岁；患者多数无明显临床症状（健康体检发现7例，咳嗽咳痰2例）；病史时间4天-3年（中位数12个月）；病变均为周围型肺结节病灶，直径0.8 cm-2.8 cm，平均（1.4±0.6）cm，所有病例均经螺旋计算机断层扫描（computed tomography, CT）检查，病变直径小于3 cm，完全被肺实质包绕，无胸膜受累，不伴有淋巴结病变、肺炎、肺不张；纵隔淋巴结无肿大。术前检查包括胸部CT、胸部X光、心电图、肺功能、血气分析等检查。高度怀疑肺癌者行头部CT或磁共振成像（magnetic resonance imaging, MRI）、骨发射单光子计算机断层扫描仪（emission computed tomography, ECT）扫描等检查；术前均未能获得病理诊断。1例术前行正电子发射断层扫描-计算机断层扫描（positron emission tomography-computed tomography, PET-CT）检查，病灶为高代谢，提示肺癌可能性大。病变位于右肺上叶2例，右肺中叶2例，右肺下叶1例，左肺下叶2例，左肺上叶2例。排除标准包括：胸膜腔弥漫粘连增厚（局限胸膜粘连者可由机器人完成分离），肿块大于3 cm，病变靠近肺门，不能行楔形切除，心肺功能差不能耐受手术者等。

### 1.2 手术方法

#### 1.2.1 术前病灶定位

因为SPN完全被肺组织包绕，胸膜无受累，肺表面无异常（图 1）；达芬奇机器人的手臂无触觉反馈，无法感知病变的部位，因此术前精准的定位非常重要；我们采用CT引导下病灶周围肺穿刺并注射亚甲蓝溶液辅助定位；术中可清楚分辨出病变所在的蓝染区域（图 2）。

**1:**
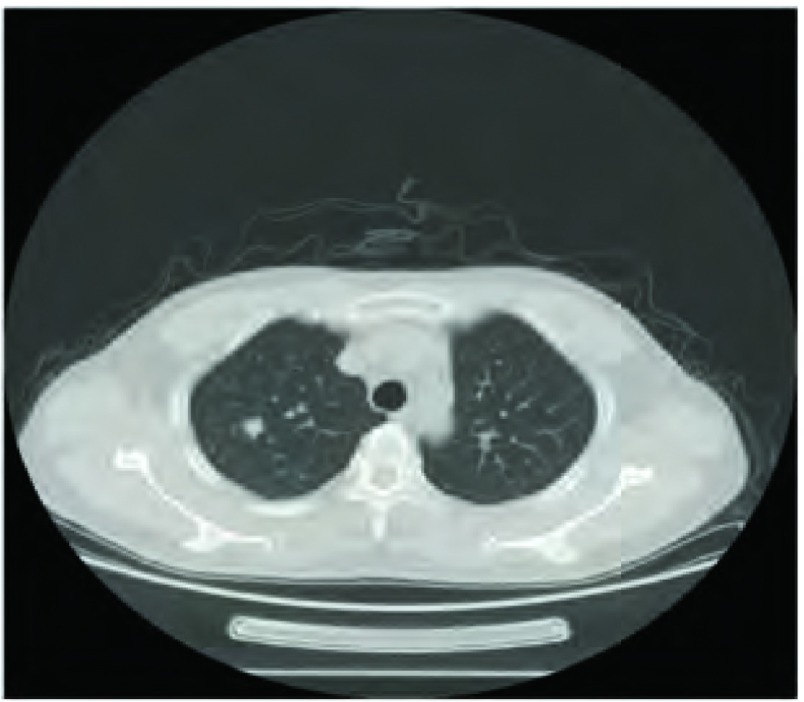
右肺上叶SPN，术后病理为腺癌 The SPN in upper lobe of right lung; histopathology: adenocarcinoma. SPN: solitary pulmonary nodule.

**2:**
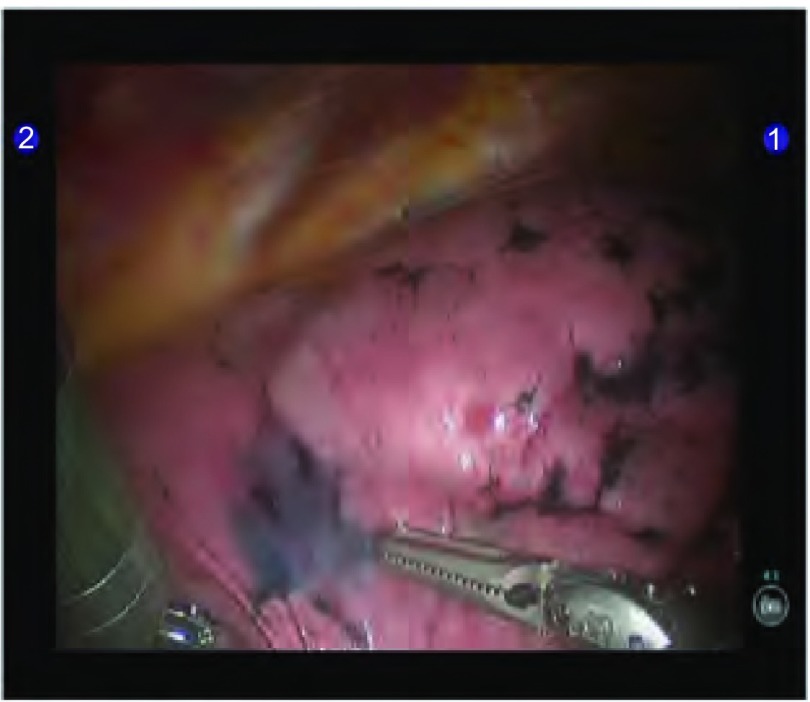
胸膜蓝染区域 Pleural blue dyeing area

#### 1.2.2 麻醉和体位

采用全麻、双腔管气管插管，健侧卧位，下胸部垫高，健侧单肺通气，双手屈曲抱枕于头前，折刀位（图 3）。

**3:**
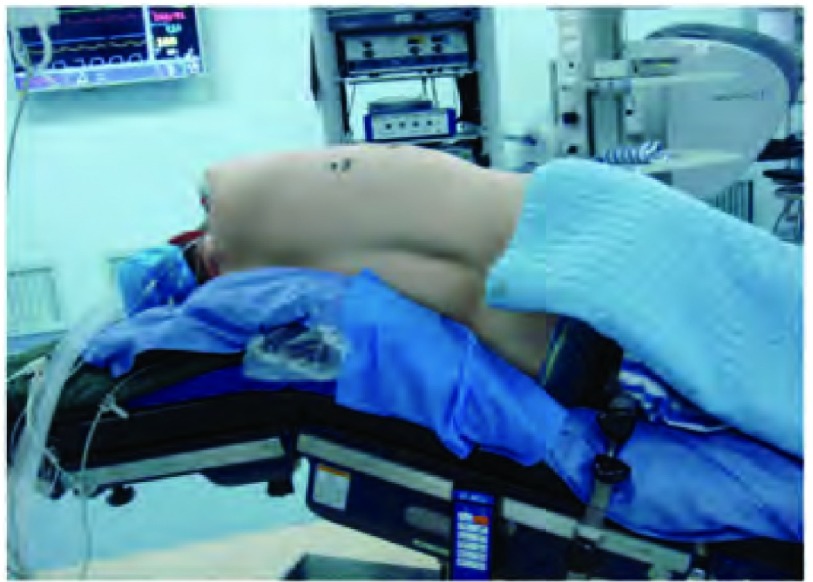
体位 Patients position

#### 1.2.3 Trocar位置和切口

孔位根据病变位置适当调整，腋后线8肋间切1.2 cm小口为进镜孔，肩胛线第8肋间、腋前线与锁中线间第5肋间各切0.8 cm小口为器械孔。腋中线第7肋间切辅助口3 cm-4 cm并用切口保护套保护（简称“8857”）。

#### 1.2.4 病变探查和切除

术中查找蓝染的区域，确定病变的部位，应用一次性直线型切割闭合器将病变楔形切除，切缘距离病变大于2 cm。病变切除后用内镜取物器将病变从辅助口取出，避免污染。病变切除后直接送冰冻病理检查，明确病变性质。

### 1.3 统计学分析

应用SPSS 19.0统计软件处理数据。

## 2 结果

本组病例无严重术中及术后并发症，无围手术期死亡。所有病例先行病灶楔形切除，其中5例冰冻病理证实为肺癌，其余4例证实为良性病变。5例肺癌患者中，右肺中叶切除+淋巴结清除术2例，左肺上叶切除+淋巴结清除术1例，其余2例患者因为心肺功能差，病变小于2 cm，未行肺叶切除，仅距病变边缘2 cm行楔形切除，并行纵隔淋巴结清除。9例均顺利完成机器人手术，手术时间60 min-190 min，平均（104.2±27.3）min。术中出血2 mL-100 mL（中位数50 mL），无输血。术后通过电话和门诊随访，随访率100%，随访时间为0.1个月-18.5个月（中位数11个月），无复发、转移。

石蜡病理及免疫组化结果：良性病变4例，其中炎性假瘤3例，错构瘤1例；恶性病变5例，均为腺癌，淋巴结均无转移；术后病理分期：Ia期5例（T1aN0M0 4例，T1bN0M0 1例）。

## 3 讨论

肺癌是我国最常见的恶性肿瘤之一，也是我国最主要的癌症致死原因之一。据预测，到2025年，我国每年将新增肺癌患者高达100万人，但令人遗憾的是，近二十年来，肺癌患者的长期生存率并没有明细的提高，其总的5年生存率仅有10%-20%；术后的非小细胞肺癌患者的术后5年生存率也仅在40%左右，但是Ⅰ期肺癌的术后5年生存率达70%-80%，因此早期发现、早期诊断及早期治疗还是肺癌治疗取得较好效果的关键。

近年来随着影像检查技术的发展和人们健康意识的提高，无症状的SPN病例逐渐增多，并随着病灶的直径的增大，恶性肿瘤的发生率也明显提高，结节直径在5 mm-10 mm时，恶性肿瘤占6%-28%；直径大于2 cm者恶性肿瘤的发生率可高达64%-82%^[2]^，这部分病例是潜在可以治愈的人群，对于这类病例给予适当的治疗是非常重要的。目前鉴别SPN良恶性的方法有以下几种：①影像学检查^[3]^中的高速螺旋CT及增强CT可以提供比以往更多的影像学证据，如分叶、毛刺、空泡征、胸膜反应、钙化及卫星灶等；但是SPN病变较小，影像学上的证据大多不明显，和术后病理的符合率较低，不能作为治疗的依据。②PET-CT检查作为近年来发展起来的诊断技术，是一种功能性的影像学诊断技术，但是在肺内病灶的良、恶性鉴别中的作用尚存在争议，最近的研究^[4]^表明在Ⅰ期肺癌中，其阳性预测值及阴性预测值分别为85%和26%左右，假阳性尤其是假阴性率较高，限制了此项技术的应用；本组1例患者术前PET-CT显示阳性结果，术后病理为炎性假瘤；因此PET-CT在SPN中的诊断意义还待进一步探讨，不能把PET-CT结果作为良恶性的判断的黄金标准。③经皮病灶活检及支气管镜穿刺活检阳性率较高，甚至可能达到100%^[5]^，但是有出血、气胸、种植转移的风险；对于阴性结果需要反复穿刺及长期随诊观察，我们认为对于能耐受手术的、位于肺周边的SPN患者，不提倡术前穿刺，应积极予以手术切除，除非对于非手术的患者，为取得明确病理诊断以指导下一步治疗，可以考虑穿刺病理检查。

本组研究9例SPN，肺癌为5例，占56%。因此对于SPN一定要引起足够的重视，对于无明确良性病变的证据的SPN，均不能排除恶性肿瘤的可能；对于随诊观察中无明显增大者也不能排除恶性肿瘤，本组1例病例观察3年，病变无明显变化，术后病理证实为肺腺癌。因此，我们认为对于SPN患者，在患者无明显手术禁忌症的情况下，应该行手术探查，以明确诊断，根据病理结果决定手术方案。

自20世纪90年代以来，胸腔镜微创技术得到广泛开展，尤其是其在肺结节病变诊断和治疗，早期肺癌的手术治疗等方面均得到满意的结果^[6]^，近年来达芬奇机器人开始应用于临床，它具有手术视野为三维立体图像，手术视野图像被放大10倍-15倍，提供真实的16:9比例的全景三维图像，具有电视胸腔镜所不具有的优势，使手术安全性大大提高。达芬奇机器人手术系统的操作手臂有腕部可自由活动的手术器械，每种器械有具体的手术任务，如夹紧、转动、缝合和组织的操纵，有7个自由度，模仿外科医生手和手腕的动作，具有比人手所具有的5个自由度更加灵活的优势，使得手术操作更加灵活、方便；该系统具有振动消除系统和动作定标系统，可保证机械臂在狭小的手术野内进行精确的操作，在微创手术方面具有比胸腔镜更大的优势^[7, 8]^。本组9例均顺利完成机器人手术，无术中大出血及副损伤，无中转开胸；手术时间60 min-190 min，术中出血2 mL-100 mL（中位数50 mL）。达芬奇机器人手术在肺手术中是安全的、可行的。

SPN完全被非组织包绕，胸膜无受累，肺表面无异常；达芬奇机器人的手臂无触觉反馈，无法感知病变的部位，因此术前精准的定位非常重要。术前定位方法有很多^[9]^，大多术前经CT定位肺病灶穿刺，通过针刺在胸膜留下创伤或染色剂，或者留置穿刺针等方法明确病变位置。我们采用CT引导下病灶周围肺穿刺并注射亚甲蓝溶液辅助定位，术中可清楚分辨出病变所在的蓝染区域。我院手术室内安置有高分辨率CT，手术当日麻醉前在手术内的CT监视下穿刺定位，然后转入手术间做术前准备，避免定位后时间过长染色剂褪色；同时由于随后即开始手术，即使穿刺造成气胸及血胸也能及时处理，不至于造成严重后果。本组9例病例，1例出现穿刺后肺表面出血，至手术时胸腔内出血量约200 mL，予以电凝止血，未发生严重后果。其余无明显并发症，术中均顺利定位并予以楔形切除病变。

本组5例肺癌中2例病例合并高血压、冠心病及糖尿病，心肺功能较差，鉴于病变小于2 cm，采用楔形切除，纵隔淋巴结清除术。尽可能的缩短手术时间，减小手术创伤，术后顺利恢复，无明显并发症。有文献^[10]^报道对于2 cm以下的早期肺癌，在切缘距离肿瘤2 cm以上的局部切除能取得和肺叶切除一样的远期效果。本组随访时间0.1个月-18.5个月（中位数11个月），未见肿瘤复发和转移，远期效果待随访观察。
